# Model-Based and Model-Free Analyses of the Neural Correlates of Tongue Movements

**DOI:** 10.3389/fnins.2020.00226

**Published:** 2020-03-24

**Authors:** Peter Sörös, Sarah Schäfer, Karsten Witt

**Affiliations:** ^1^Neurology, School of Medicine and Health Sciences, University of Oldenburg, Oldenburg, Germany; ^2^Research Center Neurosensory Science, University of Oldenburg, Oldenburg, Germany

**Keywords:** tongue, motor control, cortex, cerebellum, speech production, swallowing, functional magnetic resonance imaging, independent component analysis

## Abstract

The tongue performs movements in all directions to subserve its diverse functions in chewing, swallowing, and speech production. Using task-based functional MRI in a group of 17 healthy young participants, we studied (1) potential differences in the cerebral control of frontal (protrusion), horizontal (side to side), and vertical (elevation) tongue movements and (2) inter-individual differences in tongue motor control. To investigate differences between different tongue movements, we performed voxel-wise multiple linear regressions. To investigate inter-individual differences, we applied a novel approach, spatio-temporal filtering of independent components. For this approach, individual functional data were decomposed into spatially independent components and corresponding time courses using independent component analysis. A temporal filter (correlation with the expected brain response) was used to identify independent components time-locked to the tongue motor tasks. A spatial filter (cross-correlation with established neurofunctional systems) was used to identify brain activity not time-locked to the tasks. Our results confirm the importance of an extended bilateral cortical and subcortical network for the control of tongue movements. Frontal (protrusion) tongue movements, highly overlearned movements related to speech production, showed less activity in the frontal and parietal lobes compared to horizontal (side to side) and vertical (elevation) movements and greater activity in the left frontal and temporal lobes compared to vertical movements (cluster-forming threshold of *Z* > 3.1, cluster significance threshold of *p* < 0.01, corrected for multiple comparisons). The investigation of inter-individual differences revealed a component representing the tongue primary sensorimotor cortex time-locked to the task in all participants. Using the spatial filter, we found the default mode network in 16 of 17 participants, the left fronto-parietal network in 16, the right fronto-parietal network in 8, and the executive control network in four participants (Pearson's *r* > 0.4 between neurofunctional systems and individual components). These results demonstrate that spatio-temporal filtering of independent components allows to identify individual brain activity related to a specific task and also structured spatiotemporal processes representing known neurofunctional systems on an individual basis. This novel approach may be useful for the assessment of individual patients and results may be related to individual clinical, behavioral, and genetic information.

## Introduction

The human tongue is a unique muscular and sensory organ with critical roles in several motor tasks, such as chewing, swallowing, respiration, and speech (Sawczuk and Mosier, 2001; Hiiemae and Palmer, 2003), in addition to its somatosensory (Pardo et al., 1997; Sakamoto et al., 2010) and gustatory functions (Kobayakawa et al., 2005; Hummel et al., 2010).

To subserve its distinct motor tasks, the tongue contains intrinsic and extrinsic muscle fibers (Schumacher, 1927; Abd-El-Malek, 1939), which are extensively interwoven (Gaige et al., 2007). Intrinsic fibers originate and insert within the tongue itself, while extrinsic fibers are attached to bony structures, such as the mandible, hyoid bone, or styloid process (Sanders and Mu, 2013). This complex biomechanical architecture is the basis for the tongue's ability to move and alter its shape in all three dimensions (Kier and Smith, 1985). Moreover, adult human tongues, compared to the tongues of other mammals, are characterized by a higher proportion of slow-twitch (type I) muscle fibers, which are associated with fine motor control (Sanders et al., 2013). Intrinsic and extrinsic tongue muscles are innervated by the lateral and medial divisions of the hypoglossal nerve (cranial nerve XII), with different components of the musculature being supplied by different hypoglossal branches (Mu and Sanders, 2010) and controlled by distinct hypoglossal subnuclei (McClung and Goldberg, 2002).

The cortical and subcortical control of tongue movements has been studied thoroughly in animals and humans using various invasive and non-invasive techniques. These electrophysiologic, neuroimaging, and lesion studies suggest that voluntary (e.g., speech-related) and semi-automatic (e.g., swallowing-related) tongue movements (Martin et al., 1997) are controlled by the lateral primary sensorimotor cortex (Takai et al., 2010), supplementary motor area, basal ganglia, and cerebellum (Corfield et al., 1999; Shinagawa et al., 2003; Martin et al., 2004; Watanabe et al., 2004).

Using functional magnetic resonance imaging (FMRI), researchers investigated (1) isolated voluntary tongue movements, such as frontal (protrusion) (Arima et al., 2011), horizontal (side to side) (Riecker et al., 2000), and vertical (elevation) tongue movements (Martin et al., 2004) and (2) tongue movements as part of speaking (Riecker et al., 2005; Sörös et al., 2006), singing (Ozdemir et al., 2006; Jungblut et al., 2012), and swallowing (Sörös et al., 2009; Lowell et al., 2012). A detailed comparison of the neural correlates of different tongue movements in all three directions has not been performed yet [but see the study by Watanabe et al. (2004), comparing tongue protrusions in different directions with tongue retraction]. Moreover, almost all FMRI studies on tongue movements present only group analyses [one notable exception is the study by Martin et al. (2004), Table 5, presenting individual brain activation in all studied participants]. Finally, almost all FMRI studies on tongue motor control have been performed on older scanner hardware and with relatively small sample sizes. Our literature review was based on a systematic search of the PubMed^1^ and Google Scholar^2^ databases with the search terms “tongue FMRI” or “tongue functional magnetic resonance imaging.” The detailed presentation of this systematic review is beyond the scope of the present study.

The first aim of the present study was to identify and compare brain activity associated with different tongue movements. Following previous research, we simplified the wide range of different tongue movements and shapes and only studied movements along the three main axes of the body: frontal (protrusion) tongue movements, horizontal (side to side) tongue movements, and vertical (elevation) tongue movements. These tongue movements are used in different tongue motor tasks, primarily during chewing, swallowing, and speaking (Sawczuk and Mosier, 2001; Hiiemae and Palmer, 2003; Ferrand, 2018). Frontal (protrusion) tongue movements are almost exclusively used in speech production and singing, e.g., during the production of dental consonants (Ladefoged and Maddieson, 1996). Horizontal (side to side) tongue movements are used during chewing to position the food in the oral cavity and to form the bolus in preparation for swallowing (Hiiemae and Palmer, 2003). Vertical (elevation) tongue movements, finally, are used in both speech production [e.g., during the production of high vowels (Ladefoged and Maddieson, 1996)] and the oral phase of swallowing (Hiiemae and Palmer, 2003).

The second aim of this study was to investigate inter-individual similarities and differences in tongue movement-related brain activity. The number of swallows (Rudney et al., 1995) and of words produced per day (Mehl et al., 2007) varies considerably between individuals. Moreover, the biomechanics of articulation (Weirich et al., 2013) and of swallowing (Kennedy et al., 2010) is characterized by substantial inter-individual variability. To investigate individual brain activity, we performed an independent component analysis (ICA) of individual task-based FMRI data sets. ICA decomposes four-dimensional data into spatial maps and associated time courses (Beckmann et al., 2006). Compared to a model-based analysis, an advantage of ICA is the ability to detect unknown, not necessarily time-locked brain activity in FMRI data. This is of particular importance for experimental designs, in which the exact timing of events cannot be recorded (McKeown et al., 1998a; Calhoun et al., 2002). Moreover, ICA is able to separate brain activity from noise, such as artifacts induced by head motion or physiological processes (McKeown et al., 1998b). With a novel approach, spatio-temporal filtering of independent components, we identified individual components whose time courses were highly correlated with the expected brain response and whose spatial patterns were highly correlated with one of the established neurofunctional networks described by Smith et al. (2009).

Investigating differences in tongue motor control between different movements and across individuals is expected to deepen our knowledge not only of physiological but also of pathological tongue movements. The ultimate goal of this line of research is to understand the disruption of the supranuclear control of speech- and swallowing-related tongue movements in diseases, such as amyotrophic lateral sclerosis (ALS, Kollewe et al., 2011; Shellikeri et al., 2016) and Parkinson's disease (Van Lieshout et al., 2011) on an individual, personalized basis.

## Methods

### Participants

Twenty young healthy individuals have been investigated for the present study. Three participants were excluded from the data analysis (two because of excessive head motion, one because of widespread, most likely artifactual signal increase in the first-level analysis of the horizontal tongue movement condition). For the final data analysis, the MRI data of 17 participants (nine women, eight men) have been investigated. Mean age ± standard deviation (SD) of included participants was 25.9 ± 3.3 years (minimum: 20, maximum: 34 years). All participants met the following criteria: (1) no history of neurological disorders (such as dementia, movement disorder, stroke, epilepsy, multiple sclerosis, traumatic brain injury, migraine), psychiatric disorders (such as schizophrenia or major depression), or cancer, (2) no impaired kidney or liver function, (3) no use of psychotropic medication (in particular, antidepressants, antipsychotics, benzodiazepines, and opioids), (4) no substance abuse, (5) no excessive head motion (<1 mm relative mean displacement and <3 mm absolute mean displacement) during FMRI. Handedness was determined with the Edinburgh Handedness Inventory–Short Form (Veale, 2014). Right handedness was present in 13 individuals (handedness scores: 62.5–100), mixed handedness was found in four individuals (handedness scores: 33.3–50). Seven participants have had an MRI scan before for diagnostic or research purposes, 10 participants have never been within an MRI scanner. All data sets presented here have been acquired for this study and have not been analyzed or published previously. All participants were students of the University of Oldenburg and were recruited through an advertisement on the University's student portal or word-of-mouth communication, thus representing a convenience sample. All participants gave written informed consent for participation in the study. A compensation of 10 € per hour was provided. The study was approved by the Medical Research Ethics Board, University of Oldenburg, Germany (2017-072).

### Experimental Paradigm and Tongue Movements

The data for the present study were collected as part of a larger project on oral and speech-language functions. Table 1 summarizes the order in which the four different tasks were performed. The total MRI measurement time was ~45 min.

**Table 1:** Structural and functional sequences used for the entire project (scan 4: tongue movements).

**No**.	**Sequence**	**Time of acquisition**
1	T1-weighted MPRAGE	6:16 min
2	T2^*^-weighted (syllable production)	9:16 min
3	T2^*^-weighted (tongue twister)	8:31 min
4	T2^*^-weighted (tongue movements)	9:21 min
5	T2^*^-weighted (sentence production)	9:14 min

**Figure 1:**
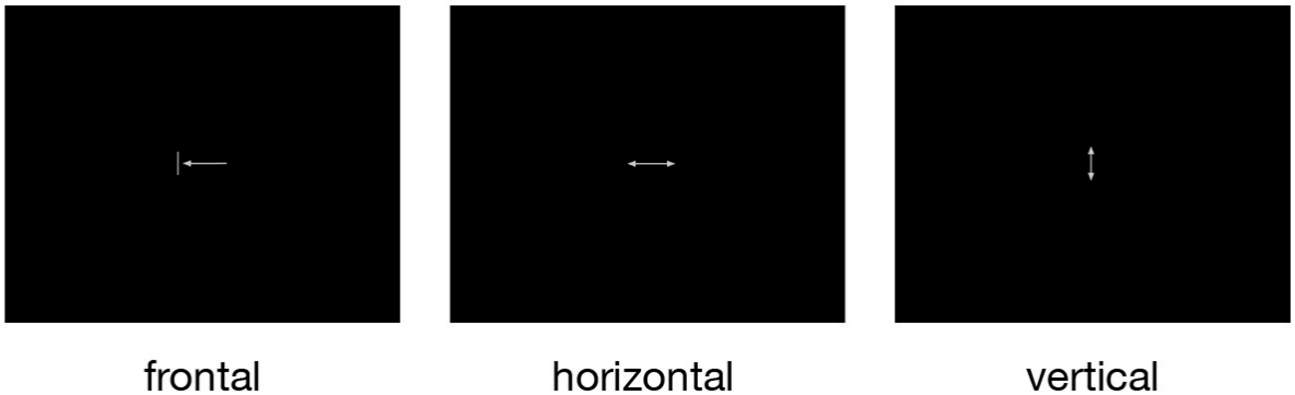
The three pictograms used to cue frontal (protrusion), horizontal (side to side), and vertical (elevation) tongue movements. All symbols were simple and small to minimize eye movements and visual processing. During rest periods, a small fixation cross was shown.

For the investigation of the neural correlates of tongue movements, participants were visually cued to perform one of three different repetitive tongue movements (Figure 1): (1) frontal (protrusion) tongue movements; participants were instructed to push the tip of the tongue against the surface of the maxillary incisors and then retract the tongue to the rest position, similar to the English /th/ sound, (2) horizontal (side to side) tongue movements; participants were instructed to move the tongue against the right and left mandibular pre-molars, and (3) vertical (elevation) tongue movements; participants were instructed to elevate the tongue and to press it against the hard palate (the roof of the mouth), similar to the beginning of the oral phase of swallowing (Dodds, 1989).

Before the FMRI measurement, all participants underwent a short training outside the scanner to familiarize themselves with the different tongue movements and the visual cues. During the training, the participants were first shown a sheet of paper with the three visual cues (Figure 1) and were instructed how to perform the respective movement. The experimenter then demonstrated the required movements herself with open mouth and asked the participants to perform all movements with closed mouth, without performing head or jaw movements. The experimenter watched all participants closely and made sure that no visible head or jaw movements were produced. For all conditions, participants performed rhythmic and self-paced movements. Participants were asked to choose a relaxed and pleasant movement frequency. Our aim was to keep the movement effort comparable across participants. Finally, a short version of the FMRI paradigm (two blocks of 15 s duration for each tongue movement in pseudorandomized order) was presented on the screen of a PC to give the participants further opportunity to train the required tongue movements.

**Figure 2:**
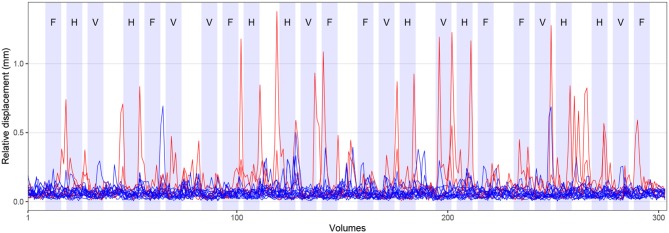
Experimental paradigm and head motion. Blocks of tongue movement are shown in light blue; F: frontal (protrusion), H: horizontal (side to side), and V: vertical (elevation) movements; duration 15 s each. Mean relative displacement, the distance between one volume and the following volume, during all FMRI measurements is displayed with blue (17 included participants) and red lines (three excluded participants). Data from individual participants are superimposed.

Visual cues were presented by the MATLAB toolbox Cogent Graphics (developed by John Romaya, Laboratory of Neurobiology, Wellcome Department of Imaging Neuroscience, London, UK)^3^ on a PC and projected through an LCD projector onto a screen mounted within the scanner bore behind the head coil. Participants were able to see the cues via a mirror attached to the head coil. Visual cues were shown in blocks of 15 s duration with a 15 s rest condition (during which a fixation cross was presented) after every third tongue movement block. The remaining tongue movement blocks were separated by a shorter rest condition of 3 s. Figure 2 visualizes the experimental paradigm. Behavioral performance during FMRI was not recorded.

### MRI Data Acquisition

MR images of the entire brain were acquired at 3 Tesla on a Siemens MAGNETOM Prisma whole-body scanner (Siemens, Erlangen, Germany) with the XR gradient system (gradient strength: 80 mT/m, gradient rise time: 200 T/m/s on all three gradient axes simultaneously) and a 64-channel head/neck receive-array coil. This coil enhances the signal-to-noise ratio of the peripheral image, primarily corresponding to the cortex of the human brain. The scanner is located at the Neuroimaging Unit, School of Medicine and Health Sciences, University of Oldenburg, Germany^4^.

For structural brain imaging, Siemens' 3-dimensional T1-weighted magnetization prepared rapid gradient echo (MPRAGE) sequence (Brant-Zawadzki et al., 1992) was used (TR: 2,000 ms, TE: 2.07 ms, flip angle 9°, isotropic voxel size: 0.75 × 0.75 × 0.75 mm^3^, 224 axial slices, time of acquisition: 6:16 min). For functional imaging, Siemens' ep2d_bold T2^*^-weighted gradient-echo echo-planar sequence was used (TR: 1,800 ms, TE: 30 ms, flip angle 75°, isotropic voxel size: 3 × 3 × 3 mm^3^, 33 slices, time of acquisition: 9:21 min). Structural and functional measurements used in-plane acceleration (generalized autocalibrating partial parallel acquisition, GRAPPA) with an acceleration factor of 2 (Griswold et al., 2002). Before every functional sequence, extended 3-dimensional B0 shimming and true-form B1 shimming was applied. Siemens' pre-scan normalization filter was deactivated during functional sequences.

### MRI Data Analysis

#### Pre-processing of Structural Images

Pre-processing of T1-weighted images was done with the *antsBrainExtraction.sh* script, part of Advanced Normalization Tools (ANTs, version 2.1)^5^ (Tustison et al., 2014). This script performs (1) bias field correction to minimize the effects of magnetic field inhomogeneity using the N4 algorithm (Tustison et al., 2010) and (2) brain extraction using a hybrid segmentation/template-based strategy (Tustison et al., 2014). The script was used together with brain templates derived from the OASIS-1 study^6^. For quality control, all brain extracted structural MR images were visually inspected after using ANTs.

#### Pre-processing of Functional Images

Pre-processing of FMRI data was carried out using FEAT (version 6.00), part of FMRIB's Software Library (FSL)^7^ (Smith et al., 2004; Woolrich et al., 2009; Jenkinson et al., 2012). Pre-processing included removal of the first four recorded volumes to allow for signal equilibration in addition to the two dummy volumes measured, but not recorded, as part of the ep2d_bold sequence (304 volumes were retained). Standard head motion correction was performed by volume-realignment to the middle volume using MCFLIRT (Jenkinson et al., 2002). Mean relative displacement (the distance between one volume and the following volume) of all participants is shown in Figure 2. Brain extraction of functional images was done with FSL's brain extraction tool, BET (Smith, 2002). Spatial smoothing with a Gaussian kernel of 5 mm full width at half maximum (FWHM) and grand-mean intensity normalization of the entire data set by a single multiplicative factor were also performed. Slice time correction was not conducted (as e.g., in Beckmann et al., 2006).

After completion of standard data pre-processing, but without temporal filtering, ICA-based automatic removal of motion artifacts (FSL's ICA-AROMA version 0.3 beta)^8^ was used to identify and remove motion-related ICA components from FMRI data (Pruim et al., 2015). Here, the non-aggressive option was used, performing a partial component regression. First, ICA-AROMA carries out probabilistic ICA of individual subjects' MRI data using FSL's MELODIC (Beckmann and Smith, 2004). Second, ICA-AROMA employs four theoretically motivated temporal and spatial criteria to select motion-related components from MELODIC's output. These criteria include (1) high-frequency content of the time courses of independent components, (2) correlation between the time courses of independent components and motion correction parameters, (3) representation of independent components at the edge of the brain, and (4) representation of independent components within cerebrospinal fluid (for a detailed description, see Pruim et al., 2015). Finally, ICA-AROMA removes these components from the initial data set through an ordinary least squares regression using FSL's *fsl_regfilt* command (Pruim et al., 2015). Decomposition of individual data sets created between 47 and 68 independent components (mean: 60 components). To determine the optimal number of components for every data set, MELODIC uses Bayesian principal component analysis (Beckmann and Smith, 2004). Of these components, between 18 and 39 (mean: 30) components were identified as noise and regressed out applying the aforementioned four temporal and spatial features. ICA-AROMA has been validated for resting state and task-based FMRI data, demonstrating that this approach effectively removes motion artifacts, while increasing sensitivity to the signal of interest (Pruim et al., 2015).

Following ICA-AROMA, data were high-pass filtered (Gaussian-weighted least-squares straight-line fitting, sigma = 45 s). Registration of functional to high-resolution structural images was carried out using FLIRT (Jenkinson et al., 2002). Registration from high-resolution structural to Montreal Neurological Institute (MNI152) standard space was further refined using 12-parameter affine transformation and non-linear registration with a warp resolution of 10 mm in FNIRT^9^.

#### Model-Based Individual and Group FMRI Analysis

For first-level model-based analysis, functional data sets were analyzed with a general linear model-based time-series analysis using voxel-wise multiple linear regressions (Friston et al., 1995; Monti, 2011) as implemented in FEAT. The time courses of the three movement conditions were convolved with a gamma hemodynamic response function (using the standard settings: phase: 0 s, standard deviation: 3 s, mean lag: 6 s) and served as regressors of interest. The temporal derivative of each primary regressor was included as a regressor of no interest to improve the model fit when the timing was not exactly correct (e.g., if tongue movements were started or stopped with a slight delay). Regressors of interest (experimental conditions) and regressors of no interest (temporal derivatives) formed the design matrix used for voxel-wise multiple linear regressions. Motion parameters derived from initial head motion correction via volume-realignment were not included in the design matrix because ICA-AROMA was used for additional head motion correction.

To remove temporal autocorrelations, time-series pre-whitening was used (Woolrich et al., 2001). After generating parameter estimates (PEs) for every primary regressor and every participant, the following contrasts of parameter estimates (COPEs) were calculated: (1) frontal > rest, (2) horizontal > rest, (3) vertical > rest, (4) horizontal > frontal, (5) vertical > frontal, (6) frontal > vertical, (7) horizontal > vertical, (8) frontal > horizontal, and (9) vertical > horizontal.

For higher-level analysis, mixed-effects group analysis maps were generated by FLAME (stages 1 and 2) for all contrasts. FLAME uses a fully Bayesian inference technique in a two-stage process: a fast approach using maximum *a posteriori* estimates and a slower, more accurate approach using Markov Chain Monte Carlo methods (Woolrich et al., 2004). *Z* statistic images were thresholded non-parametrically using a cluster-forming threshold of *Z* > 3.1 and a (corrected) cluster significance threshold of *p* < 0.01 assuming a Gaussian random field for the *Z*-statistics. No additional correction for multiple contrasts was performed. Local maxima (peaks of brain activation) were identified within the *Z* statistic images using FSL's *cluster* command (maximum number of local maxima: 100, minimum distance between local maxima: 20 mm). The anatomical location of each local maximum was determined with FSL's *atlasquery* command and the following probabilistic atlases:^10^ (1) Harvard-Oxford cortical structural atlas (48 cortical areas), (2) Harvard-Oxford subcortical structural atlas (21 subcortical areas), and (3) Probabilistic cerebellar atlas (28 regions) (Diedrichsen et al., 2009). Because we report local maxima of brain activation, the extent of activation cannot be determined.

#### Model-Free Individual FMRI Analysis

A single-session probabilistic ICA was conducted to decompose every pre-processed individual FMRI data set into 20 independent spatial components and corresponding time courses using MELODIC (version 3.14). The data sets fed into ICA were the pre-processed data sets used for first-level model-based analysis (i.e., after denoising with ICA-AROMA). Of note, these data sets contained brain activity associated with all three tongue movements. A low-dimensional decomposition into 20 components was chosen for later comparison with a well-established set of published networks. MELODIC performs linear decomposition (Comon, 1994) of the original FMRI signal using the FastICA technique (Hyvärinen, 1999) and variance-normalization of the associated timecourses. Spatial maps were thresholded using a Gaussian mixture model approach at a posterior probability level of *p* < 0.5 (Beckmann and Smith, 2004).

#### Spatio-Temporal Filtering of Independent Components

To identify spatial components whose activity was time-locked to tongue movements, FSL's GLM Setup program was used to convolve the time course of all three movement conditions with a gamma hemodynamic response function (as for model-based analysis). The resulting time course, representing the expected brain activity, was correlated with all ICA time courses of all participants. Similarly, Kokkonen et al. (2009) analyzed task-based FMRI activity associated with left and right finger tapping with ICA and correlated ICA time courses with motor task timing. In our study, a significant (*p* < 0.05) Pearson's correlation coefficient of *r* > 0.4 or *r* < −0.4 between time courses indicated temporal significance.

To identify relevant spatial components not time-locked to the tongue motor tasks, the spatial maps obtained for every participant were cross-correlated with ten established neural networks^11^ (Smith et al., 2009). These ten networks have been derived from resting state FMRI data of healthy adults after performing a low-dimensional decomposition with 20 components (ten components were identified as brain activity, ten components as potentially artifactual). Importantly, Smith et al. (2009) also analyzed the results of 1687 task-based FMRI studies available through the BrainMap database^12^. Both analyses resulted in a similar set of major brain networks, demonstrating a close correspondence between resting and task-based brain activation. A significant Pearson's correlation coefficient of *r* > 0.4 between components indicated spatial significance. The cross-correlation of study-specific spatial components with established networks (e.g., developed by Smith et al., 2009 or Yeo et al., 2011) has been performed previously for the analysis of resting-state FMRI data (e.g., by Reineberg et al., 2015 and Sörös et al., 2019).

## Results

### Head Motion

Across all included participants, the average mean absolute displacement of the head (relative to the middle volume) was 0.7 mm (SD: 0.4 mm, min: 0.2 mm, max: 1.9 mm). The average mean relative displacement (compared to the following volume) was 0.3 mm (SD: 0.2 mm, min: 0.1 mm, max: 0.7 mm). The time course of the mean relative displacement of all participants is shown in Figure 2 (17 included participants in blue, three excluded participants in red). Comparing the time course of head displacement with the experimental paradigm, we did not find evidence for task-related head motion.

### Model-Based Group FMRI Analysis

**Figure 3:**
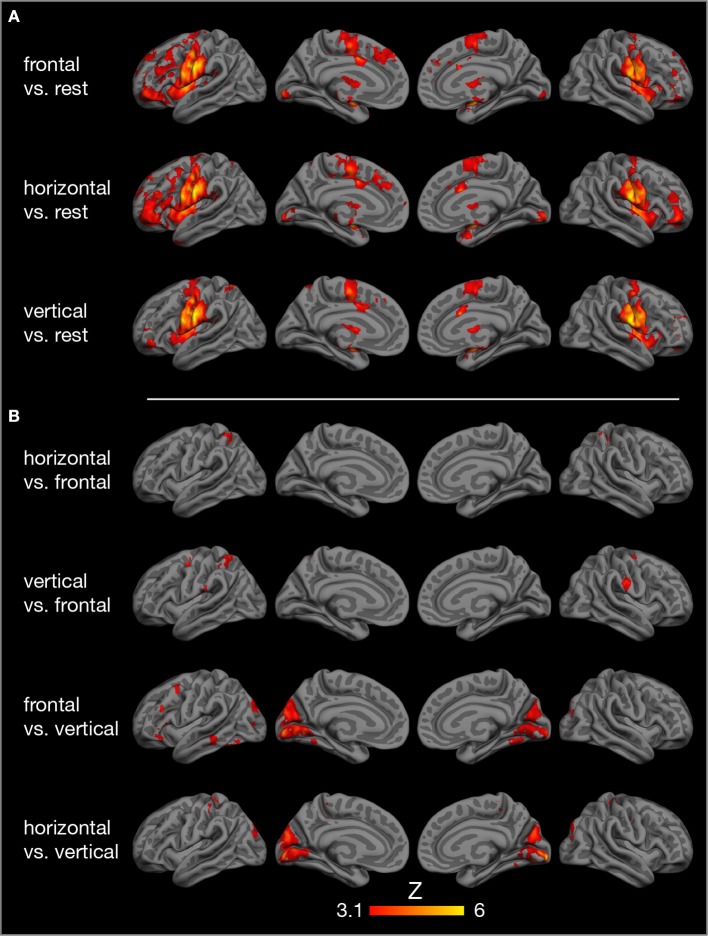
Results of model-based group analysis. Brain activity associated with tongue movements averaged across 17 participants after cluster-based thresholding and correction for multiple comparisons (*Z* > 3.1, *p* < 0.01) is shown in red-yellow. Activated areas were projected onto the semi-inflated pial surface of the *fsaverage* brain, reconstructed by FreeSurfer (Fischl, 2012). **(A)** Tongue movements > rest, **(B)** contrasts between tongue movements.

**Figure 4:**
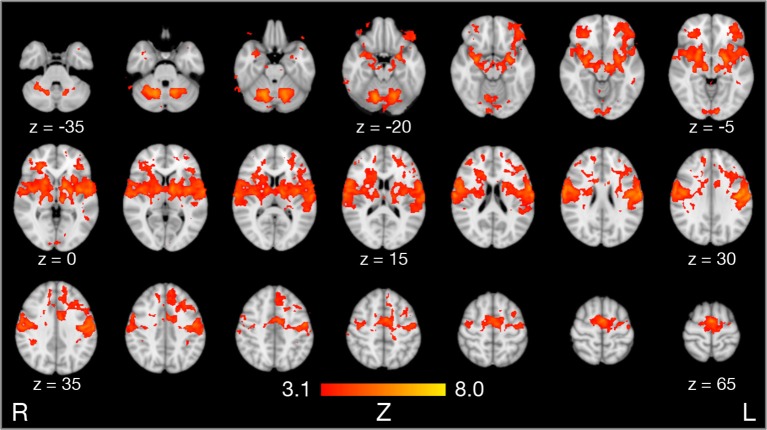
Results of model-based group analysis. Brain activity associated with frontal tongue movements vs. rest averaged across 17 participants after cluster-based thresholding and correction for multiple comparisons (*Z* > 3.1, *p* < 0.01) is shown in red-yellow. Brain activity is projected onto axial slices of the MNI152 standard space template in radiological convention (the left hemisphere is seen on the right).

Compared to rest, the three tongue movements were associated with similar bilateral brain activity (Figure 3A). Major regions of cortical activation were the lateral pre- and post-central gyrus, supplementary motor cortex, anterior cingulate gyrus, and the frontal cortex of both hemispheres. Activation was also found in the bilateral insulae, basal ganglia, thalamus, amygdala, and cerebellum. For visualization of subcortical brain activity, Figure 4 shows axial slices for frontal (protrusion) tongue movements > rest. Table 2 lists the coordinates in MNI space and the corresponding *Z* value of significant local maxima of brain activation for the contrast frontal > rest.

**Table 2:** Local maxima in brain activation: stereotaxic coordinates in MNI space, *Z* values, and corresponding brain regions for the contrast frontal tongue movement > rest.

**Region**	**Side**	***x* (mm)**	***y* (mm)**	***z* (mm)**	***Z* value**
Precentral gyrus	L	−60	2	20	6.24
	R	58	8	8	5.00
	R	42	−10	52	4.56
Supplementary motor cortex		0	−4	64	6.22
Superior frontal gyrus		0	32	46	6.00
	L	−12	16	56	5.03
Middle frontal gyrus	L	−50	16	38	4.36
	L	−26	20	38	5.31
Inferior frontal gyrus, pars triang.	L	−48	28	16	5.14
Frontal operculum	R	34	16	10	4.65
Frontal orbital cortex	L	−34	18	−12	5.02
Frontal pole	R	36	42	20	4.51
Post-central gyrus	L	−42	−16	32	6.56
	R	62	−6	28	6.17
Supramarginal gyrus, post. div.	L	−38	−50	42	4.49
	R	34	−38	36	5.44
Central opercular cortex	L	−60	−20	12	5.78
Middle temporal gyrus	L	−54	−54	0	3.97
Lingual gyrus	L	−14	−78	−2	3.94
	R	6	−92	−16	6.76
Cingulate gyrus, ant. div.	L	−8	1	37	4.65
	R	6	0	36	3.53
Insular cortex	L	−40	−4	2	5.81
	R	42	−4	4	5.21
Putamen	R	22	8	−2	5.51
Pallidum	L	−13	4	0	5.63
Thalamus	L	−10	−2	4	5.93
	R	14	−4	8	5.45
Amygdala	L	−26	−4	−16	5.77
	R	28	−2	−16	5.65
Cerebellum lobule VI	L	−16	−64	−22	6.08
	R	16	−66	−22	6.60

Contrasts between different tongue movements resulted in significant activation in several cortical areas (Figure 3B, Table 3). Horizontal tongue movements were associated with greater activation in the bilateral superior parietal lobule (vs. frontal movements) and in the bilateral pre- and post-central gyri as well as in the left inferior frontal gyrus (vs. vertical movements). The contrast vertical > frontal movements demonstrated greater activation in the bilateral precentral and the right post-central gyri, as well as the left supramarginal gyrus and the left superior parietal lobule. The reverse contrast, frontal > vertical movements, showed greater activation in the left frontal and temporal lobes. The contrasts frontal > horizontal and vertical > horizontal did not result in significant activation.

**Table 3:** Local maxima in brain activation: stereotaxic coordinates in MNI space, *Z* values, and corresponding brain regions for the contrasts horizontal > frontal, vertical > frontal, frontal > vertical, and horizontal > vertical tongue movements.

**Region**	**Side**	***x* (mm)**	***y* (mm)**	***z* (mm)**	***Z* value**
**Horizontal > frontal**					
Superior parietal lobule	L	−32	−50	58	4.75
	R	26	−48	66	4.98
**Vertical > frontal**					
Precentral gyrus	L	−26	−12	62	5.29
	R	32	−6	66	4.59
Post-central gyrus	R	52	−20	34	6.70
	R	32	−34	36	4.67
Central opercular cortex	L	−60	−20	16	4.72
Supramarginal gyrus, ant. div.	L	−44	−34	38	5.03
Superior parietal lobule	L	−34	−48	52	4.69
**Frontal > vertical**					
Middle frontal gyrus	L	−46	10	46	4.56
Inferior frontal gyrus, pars triang.	L	−46	24	12	4.95
Frontal orbital cortex	L	−42	30	−16	4.29
Middle temporal gyrus, post. div.	L	−60	−36	−10	4.33
Inferior temporal gyrus, temp-occ.	L	−42	−50	−16	4.47
Cuneal cortex	R	2	−78	22	5.56
Lateral occipital cortex	L	−20	−86	20	4.79
	R	28	−82	12	4.85
Intracalcarine cortex	R	8	−64	8	4.45
Lingual gyrus	L	−10	−78	0	5.35
Occipital pole		0	−96	−4	6.33
	R	24	−96	−8	3.87
Occipital fusiform gyrus	L	−32	−72	−12	4.29
	R	26	−66	−6	4.97
**Horizontal > vertical**					
Precentral gyrus	L	−22	−26	58	4.74
	L	−40	−16	46	4.60
	R	22	−28	68	4.97
Inferior frontal gyrus, pars triang.	L	−54	28	−10	4.53
Post-central gyrus	L	−2	−38	58	4.83
	R	38	−26	50	4.67
Cingulate gyrus, post. div.	L	−2	−20	44	4.23
Cuneal cortex	L	−6	−88	22	5.05
	R	10	−70	20	5.17
Occipital pole	L	−10	−90	−2	5.65
	R	16	−98	−6	8.61
Fusiform cortex	R	26	−60	−16	5.16

### Model-Free Individual FMRI Analysis

**Figure 5:**
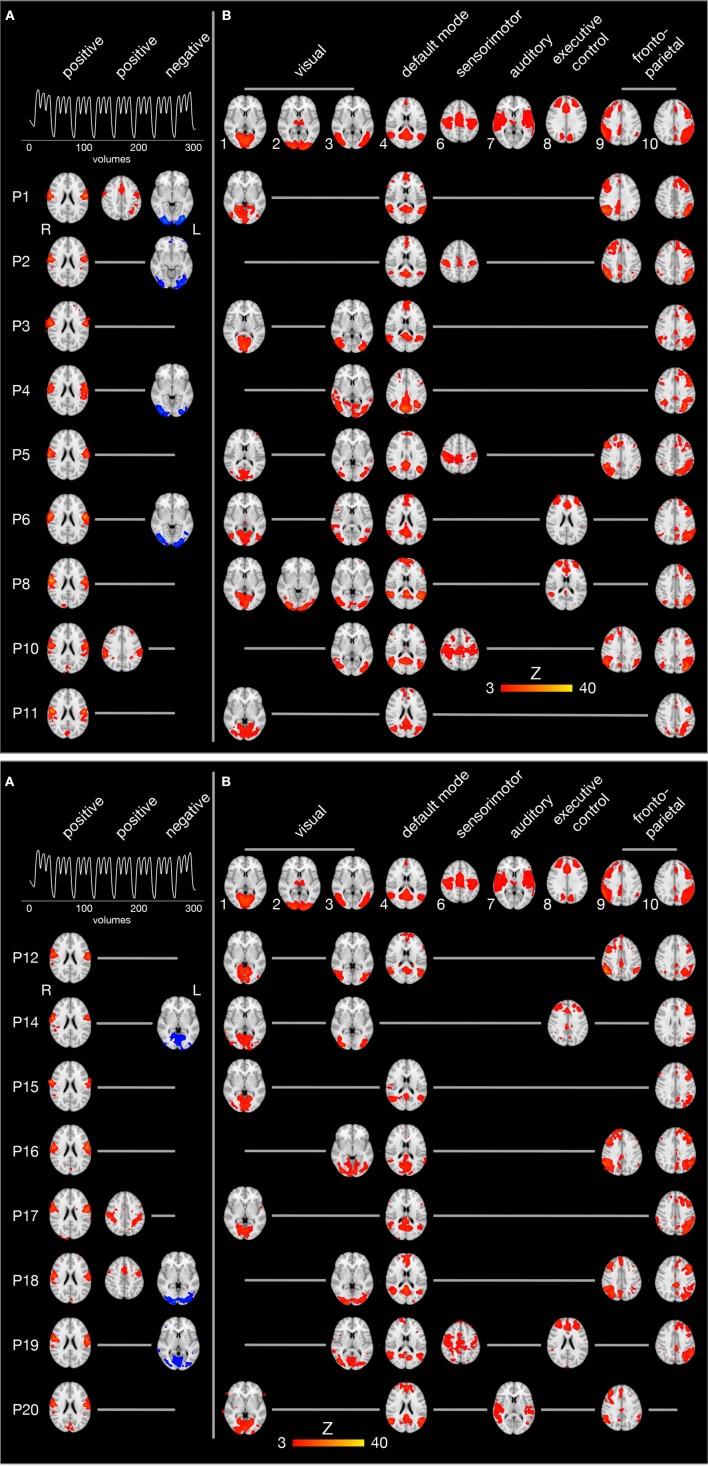
Spatio-temporal filtering of individual ICA components. The results of the single-session independent component analyses of all 17 participants (one participant per row) are summarized. Brain images are shown in radiological convention (the left hemisphere is seen on the right) after registration to the MNI152 standard space template. **(A)** The three columns on the left display components whose time course is significantly correlated with the expected brain activity (first row). The first and second column show positively correlated (*r* > 0.4, *p* < 0.05), the third column negatively correlated components (blue, *r* < −0.4, *p* < 0.05). **(B)** The upper row illustrates 9 of 10 established brain networks identified in resting state FMRI data (Smith et al., 2009). For every single participant, independent components are displayed that are spatially correlated with one of the established networks. Network 5 from the study by Smith et al. (2009) (consisting of large parts of the cerebellum) is not shown because it was not significantly correlated with cerebellar activity in one of the present FMRI data sets.

The individual brain activity of all 17 participants is presented in Figure 5. Temporally filtered (time-locked) independent components are shown on the left (Figure 5A), spatially filtered components on the right (Figure 5B). In all participants, the FMRI data set contained one bilateral sensorimotor component (Figure 5A, first column) positively correlated with the expected brain activity (graph in the first row), representing the tongue primary sensorimotor cortex. In four participants, another frontal or parietal component (Figure 5A, second column) was positively correlated with the expected brain activity. In seven participants, a bilateral occipital component (Figure 5A, third column) was negatively correlated with the expected brain activity.

Spatial filtering of independent components revealed inter-individually variable patterns of brain activity during tongue movements. Spatial cross-correlations with established neural networks (shown in the first row of Figure 5B) (Smith et al., 2009) identified between one and three visual networks in all participants (except P2, who showed a visual component after temporal filtering). In addition, the default mode network and the left fronto-parietal network were active in all but one participant. Of note, the default mode network was not negatively correlated with the expected brain activity associated with tongue movements. The right fronto-parietal network was active in eight participants and the executive control network in four participants. In four participants, a superior sensorimotor network, comprising the hand sensorimotor cortex and the supplementary motor area, was found.

## Discussion

Main results of the present task-based FMRI study on the neural correlates of tongue movements were: (1) All three tongue movements under investigation were controlled by the same neurofunctional system, consisting of the bilateral tongue primary sensorimotor cortex, supplementary motor cortex, anterior cingulate gyrus, basal ganglia, thalamus, and cerebellum. (2) Distinct tongue movements also involved more specialized regions, such as the prefrontal, posterior parietal, and temporal cortices. (3) Using a novel approach to characterize inter-individual differences in task-based FMRI data, spatio-temporal filtering of independent components, we found consistent activation of the tongue primary sensorimotor cortex in all participants, but also remarkable variability, e.g., in fronto-parietal and executive control networks.

### Model-Based Group FMRI Analysis

The present study demonstrates the core cortical [lateral primary motor cortex (Fesl et al., 2003), supplementary motor area, cingulate motor area] and subcortical regions (basal ganglia, thalamus, cerebellum) of the tongue motor system, corroborating several previous FMRI studies (Corfield et al., 1999; Shinagawa et al., 2003; Martin et al., 2004; Watanabe et al., 2004; Brown et al., 2008; Malandraki et al., 2009). The tongue motor system was very similar for all three tongue movements under investigation (Figure 3). The local maxima of precentral activation (Table 2) correspond well to activation maxima reported in the literature, e.g., by Arima et al. (2011) for tongue protrusion and by Fesl et al. (2003) for horizontal tongue movements, supporting our interpretation that the precentral brain activation seen here represents the primary tongue motor cortex. Our results also demonstrate the involvement of the lateral primary somatosensory cortex, reflecting the extensive mechanosensory (Kaas et al., 2006) and proprioceptive innervation of the tongue (Adatia and Gehring, 1971).

Moreover, the bilateral insular cortex was active during all three tongue movements. The insulae are not regarded as motor areas *per se*, but as areas of polymodal sensory, motor, cognitive, and affective integration. The insular cortex is involved in processing somatosensory (Sörös et al., 2008; Pugnaghi et al., 2011), gustatory (Small, 2010), and nociceptive stimuli (Xu et al., 2019). In addition, insular activity is associated with voluntary and semi-voluntary oro-facial movements, such as jaw opening and closing (Wong et al., 2011), speech production (Simonyan and Fuertinger, 2015; Tourville et al., 2019), and swallowing (Sörös et al., 2009; Leopold and Daniels, 2010; Malandraki et al., 2011). Importantly, insular activity is not specific for oro-facial movements, but has been found in simple finger movements as well (Turesky et al., 2016).

Tongue motor control was also associated with activity in prefrontal areas, critical for motor planning (Svoboda and Li, 2018), and in posterior parietal areas, involved in processing and perception of action-related information (Culham and Valyear, 2006). Parietal activity has also been found in previous FMRI studies during frontal tongue movements (tapping of the tip of the tongue against the alveolar ridge) (Malandraki et al., 2009) and a series of spatially complex tongue movements (pressing the inside of a left or right, upper or lower incisor, canine, or molar tooth with the tip of the tongue) (Watanabe et al., 2004).

Comparing brain activity between different tongue movements resulted in complex patterns of activation differences (Table 3). Speech-related frontal (protrusion) tongue movements were associated with less activation in parts of the bilateral superior parietal lobule (vs. horizontal, side to side movements) and in parts of the bilateral precentral gyrus, right post-central gyrus, and the left posterior parietal cortex (vs. vertical, elevation movements). We may speculate that, in most humans, speech-related tongue movements are probably some of the most overlearned movements (Ziegler, 2003) and therefore are performed with less neural resources than less extensively trained tongue movements. Similarly, complex and relatively unfamiliar sequential finger movements are associated with increased FMRI activity compared with repetitive movements of the same fingers (Wexler et al., 1997). Remarkably, frontal (protrusion) tongue movements were associated with increased activity in parts of the left frontal and temporal lobes compared with vertical (elevation) movements. Again, we may speculate that frontal (protrusion) tongue movements, usually performed in the context of overt speech production, activate areas critical to speech-language production, such as the left inferior frontal gyrus (Flinker et al., 2015), even when performed in isolation.

Unexpectedly, we found increased brain activity in the occipital lobe in the contrasts frontal vs. vertical and horizontal vs. vertical movements (Figure 3, Table 3). Because previous FMRI studies do not report occipital activity, we are reluctant to attribute this activity to tongue movement. Still, it may be possible that frontal and horizontal tongue movements, exploring the oral cavity with the densely innervated tip of the tongue, contribute to the development of oral awareness (Haggard and de Boer, 2014) and activate not only somatosensory but also visual cortices. An alternative explanation would be that the different visual stimuli used to cue the three tongue movements (Figure 1) may have induced different activation patterns in visual areas.

Overall, the differences of brain activity during the three different tongue movement conditions need to be interpreted with caution. As we do not have EMG recordings of tongue activity during FMRI, we cannot be sure that tongue movements were produced with the same velocity and pressure. Differences in motion parameters could well explain the subtle differences of brain activity seen here (Wexler et al., 1997).

### Model-Free Individual FMRI Analysis

Almost all task-based and resting-state FMRI studies only present group analyses of brain activity. Recently, individual differences in brain structure and function have attracted growing attention (Kanai and Rees, 2011; Finn et al., 2015; Dubois and Adolphs, 2016). The study of individual differences of the neural control of tongue movements is expected to improve our understanding of tongue motor impairment, e.g., in amyotrophic lateral sclerosis (Kollewe et al., 2011) or Parkinson's disease (Van Lieshout et al., 2011), and help in the assessment of the efficacy of treatment options, such as tongue motor training (Arima et al., 2011; Komoda et al., 2015).

To investigate inter-individual differences in tongue movement-related brain activity, we used a novel approach, spatio-temporal filtering of independent components. First, ICA was employed to perform a low-dimensional decomposition of every single FMRI data set of 9:21 min duration. ICA carried out a model-independent separation of the original data into components that are related to brain activity, physiological extra-cerebral processes (such as respiration or blood pulsation), and imaging artifacts (such as head motion or susceptibility artifacts). The primary advantage of a model-free ICA was the detection of previously unexpected patterns of brain activity (McKeown et al., 1998b). In the traditional general linear model-based analysis of FMRI time series, these patterns of activity would be considered as noise and discarded (Monti, 2011).

Second, we used a temporal filter to identify independent components whose time course was correlated with the expected brain response (for the use of correlation as a filter technique, see Mwangi et al., 2014). This approach demonstrated that the activation of the lateral primary sensorimotor cortex was found in all 17 participants. Tongue movement is, similar to e.g., finger tapping (Engström et al., 2004), a very robust sensorimotor paradigm.

Third, we applied a spatial filter, i.e., we cross-correlated the independent components obtained for our participants with a set of ten established components (interpreted as neural networks). These canonical networks were derived from resting state FMRI data (Smith et al., 2009). Of importance, an analysis of task-based brain activation performed for the same study identified similar networks, demonstrating a close correspondence between resting and task-based brain activation (Smith et al., 2009). A study by Calhoun et al. (2008) investigated neural networks in rest and during an auditory oddball task, finding the same 11 networks in both conditions with one additional network (anterior cingulate) only present during rest.

Nine of 10 canonical networks (Smith et al., 2009) were also found in our task-based data sets of tongue movement. In 16 participants, the default mode network was active, a large-scale neural network, including the posterior cingulate cortex, precuneus, medial prefrontal gyrus, and inferior parietal cortex (Greicius et al., 2003). In 16 participants, the left fronto-parietal network and in eight participants, the right fronto-parietal network was active. Fronto-parietal networks have been shown to subserve attentional mechanisms (Markett et al., 2014) and are probably involved in a wide variety of other tasks.

Results from spatio-temporal filtering of independent components suggest that large-scale neural networks are active during simple tongue movements. Activation of these networks is not time-locked to tongue movement and thus is not detectable in model-based FMRI analysis. Of major importance, not all established networks appear to be active in all individuals. At present, it is unclear if the pattern of neural networks seen here is specific for tongue movement or, more likely, reflecting motor control in general. Moreover, it is undetermined which factors contribute to the physiological activation or deactivation of specific networks in different individuals during tongue motor control. In addition, we do not know if the pattern of neural networks found in a single individual is stable over time or may change depending on internal states or external stimulation. Results from resting state FMRI suggest that at least the major heteromodal association networks, such as the default mode network and the fronto-parietal networks, are stable over time (Zuo and Xing, 2014).

Knowledge of the neural basis of pathological tongue movements is very limited (Mohammadi et al., 2009; Kollewe et al., 2011; Shen et al., 2015). We assume that spatio-temporal filtering of independent components may be able to determine individual patterns of brain activity related to functional impairment. Of importance, this approach may differentiate between impaired activation of the primary tongue motor cortex and disorders of large-scale association networks. For clinical use, this approach may assist in detecting disordered tongue motor control in an early stage of neurodegenerative disease and may help monitoring efficacy of pharmacological treatment, non-invasive brain stimulation, or training. Recently, resting state and task-based FMRI were used to predict a cognitive trait (fluid intelligence, reading comprehension) (Greene et al., 2018; Jiang et al., 2020). Both studies found that functional connectivity based on task-based FMRI has higher prediction performance than the results of resting state FMRI. Similarly, the results of spatio-temporal filtering of independent components may be used to predict clinical parameters and may be correlated with individual behavioral, clinical, and genetic information.

### Limitations and Directions for Future Research

Our study has limitations that need to be addressed. The sample size of our study (17 participants included in final data analysis) is relatively small, at least compared to the recommendation of *n* = 30 for a typical task-based FMRI study (Turner et al., 2018). However, our literature search identified only one FMRI study of tongue motor control with a larger sample size (*n* = 24, Watanabe et al., 2004). Nevertheless, a larger sample size would have been helpful for the robust identification of differences between tongue movements in the model-based analysis and of inter-individual differences in the model-free analysis.

We did not record tongue motion during FMRI. Using surface EMG, it is possible to record electric activity of the suprahyoid muscles and then identify different tongue movements with high accuracy using a support vector machine algorithm for pattern recognition (Sasaki et al., 2016). To ensure that the behavioral performance was as similar as possible across participants, we gave detailed instructions and performed a training session before FMRI scanning. Of note, recording EMG activity would have been helpful to refine the time course of expected brain responses for model-based analysis on an individual basis. By contrast, EMG recordings would have not been used for model-free ICA (Calhoun and de Lacy, 2017). Moreover, we did not record potential jaw movements that may have been occurred during tongue movements. Recording the opening and closing of the jaw is possible with e.g., a fiber-optic sensor attached to the chin (Sörös et al., 2010).

Our experimental paradigm included regular rest periods of 15 s duration after every third tongue movement block (Figure 2). During this rest period, the hemodynamic response curve is supposed to reach the pre-movement baseline. To keep the entire duration of the tongue movement experiment shorter than 10 min (we also acquired data for three separate speech production experiments), while still recording eight blocks of 15 s length per movement condition, we chose shorter rest periods of 3 s each between the remaining tongue movement blocks. During this shorter rest period, the hemodynamic response curve has not reached the baseline. Thus, we cannot rule out the possibility that BOLD activity from one tongue block was carried over to the next block. This may have impaired our ability to detect differences between tongue movements in model-based analysis. Of note, model-free ICA does not require specific rest periods and ICA results should not be affected by shorter rest periods.

For future research on tongue motor control, we recommend to (1) replicate our investigation of different tongue movements with a larger sample size to increase statistical power and reproducibility, (2) use different stimuli (e.g., auditory instructions) to cue tongue movements in order to determine wether certain tongue movements are associated with activity in the visual cortex, (3) record surface EMG from the suprahyoid muscles during the pre-scan training session and during FMRI, (4) study tongue motor control in younger and older individuals because disorders of tongue movement are often related to diseases prevalent in the elderly and differences in brain activation between younger and older individuals have been shown for speech motor tasks (Sörös et al., 2011; Tremblay et al., 2017), (5) correlate inter-individual differences in brain activity determined with spatio-temporal filtering of independent components with age, sex, and behavioral parameters of tongue movement in a larger sample of healthy younger and older individuals, and (6) investigate the neural correlates of tongue movements in patients with neurogenic dysarthria and dysphagia on an individual basis and correlate FMRI results with etiology, disease severity, and behavioral performance.

## Data Availability Statement

The datasets generated for this study are available on request to the corresponding author.

## Ethics Statement

The studies involving human participants were reviewed and approved by Medical Research Ethics Board, University of Oldenburg, Germany. The participants provided their written informed consent to participate in this study.

## Author Contributions

PS and SS designed the study, acquired the MRI data, and performed the data analysis. SS recruited the participants. PS prepared the figures and wrote the manuscript. SS and KW revised the manuscript and participated in the interpretations of the findings. PS conceived the spatio-temporal filtering of independent components approach.

### Conflict of Interest

The authors declare that the research was conducted in the absence of any commercial or financial relationships that could be construed as a potential conflict of interest.

## Footnotes

^1^www.ncbi.nlm.nih.gov/pubmed

^2^scholar.google.com

^3^www.vislab.ucl.ac.uk/cogent_graphics.php

^4^uol.de/en/medicine/biomedicum/neuroimaging-unit

^5^stnava.github.io/ANTs/

^6^www.oasis-brains.org

^7^fsl.fmrib.ox.ac.uk/fsl/fslwiki/

^8^github.com/maartenmennes/ICA-AROMA

^9^fsl.fmrib.ox.ac.uk/fsl/fslwiki/FNIRT

^10^fsl.fmrib.ox.ac.uk/fsl/fslwiki/Atlases

^11^www.fmrib.ox.ac.uk/datasets/brainmap+rsns/

^12^www.brainmap.org

## References

[B1] Abd-El-Malek S. (1939). Observations on the morphology of the human tongue. J. Anat. 73, 201–210. 17104752 PMC1252504

[B2] Adatia A. Gehring E. (1971). Proprioceptive innervation of the tongue. J. Anat. 110, 215–220. 4259251 PMC1271091

[B3] Arima T. Yanagi Y. Niddam D. Ohata N. Arendt-Nielsen L. Minagi S.. (2011). Corticomotor plasticity induced by tongue-task training in humans: a longitudinal fMRI study. Exp. Brain Res. 212, 199–212. 10.1007/s00221-011-2719-7 21590261

[B4] Beckmann C. Jenkinson M. Woolrich M. Behrens T. Flitney D. Devlin J.. (2006). Applying FSL to the FIAC data: model-based and model-free analysis of voice and sentence repetition priming. Hum. Brain Mapp. 27, 380–391. 10.1002/hbm.20246 16565953 PMC2653076

[B5] Beckmann C. Smith S. (2004). Probabilistic independent component analysis for functional magnetic resonance imaging. IEEE Trans. Med. Imaging 23, 137–152. 10.1109/TMI.2003.822821 14964560

[B6] Brant-Zawadzki M. Gillan G. Nitz W. (1992). MP RAGE: a three-dimensional, T1-weighted, gradient-echo sequence–initial experience in the brain. Radiology 182, 769–775. 10.1148/radiology.182.3.1535892 1535892

[B7] Brown S. Ngan E. Liotti M. (2008). A larynx area in the human motor cortex. Cereb. Cortex 18, 837–845. 10.1093/cercor/bhm131 17652461

[B8] Calhoun V. de Lacy N. (2017). Ten key observations on the analysis of resting-state functional MR imaging data using independent component analysis. Neuroimaging Clin. N. Am. 27, 561–579. 10.1016/j.nic.2017.06.012 28985929 PMC5657522

[B9] Calhoun V. Kiehl K. Pearlson G. (2008). Modulation of temporally coherent brain networks estimated using ICA at rest and during cognitive tasks. Hum. Brain Mapp. 29, 828–838. 10.1002/hbm.20581 18438867 PMC2649823

[B10] Calhoun V. Pekar J. McGinty V. Adali T. Watson T. Pearlson G. (2002). Different activation dynamics in multiple neural systems during simulated driving. Hum. Brain Mapp. 16, 158–167. 10.1002/hbm.10032 12112769 PMC6872105

[B11] Comon P. (1994). Independent component analysis, a new concept. Signal Process. 36, 287–314. 10.1016/0165-1684(94)90029-9

[B12] Corfield D. Murphy K. Josephs O. Fink G. Frackowiak R. Guz A.. (1999). Cortical and subcortical control of tongue movement in humans: a functional neuroimaging study using fMRI. J. Appl. Physiol. 86, 1468–1477. 10.1152/jappl.1999.86.5.1468 10233106

[B13] Culham J. Valyear K. (2006). Human parietal cortex in action. Curr. Opin. Neurobiol. 16, 205–212. 10.1016/j.conb.2006.03.005 16563735

[B14] Diedrichsen J. Balsters J. Flavell J. Cussans E. Ramnani N. (2009). A probabilistic MR atlas of the human cerebellum. Neuroimage 46, 39–46. 10.1016/j.neuroimage.2009.01.045 19457380

[B15] Dodds W. (1989). Physiology of swallowing. Dysphagia 3, 171–178. 10.1007/BF02407219 2700955

[B16] Dubois J. Adolphs R. (2016). Building a science of individual differences from fMRI. Trends Cogn. Sci. 20, 425–443. 10.1016/j.tics.2016.03.014 27138646 PMC4886721

[B17] Engström M. Ragnehed M. Lundberg P. Söderfeldt B. (2004). Paradigm design of sensory-motor and language tests in clinical fMRI. Neurophysiol. Clin. 34, 267–277. 10.1016/j.neucli.2004.09.006 15890160

[B18] Ferrand C. (2018). Speech Science. An Integrated Approach to Theory and Clinical Practice. Boston, MA: Pearson Education.

[B19] Fesl G. Moriggl B. Schmid U. Naidich T. Herholz K. Yousry T. (2003). Inferior central sulcus: variations of anatomy and function on the example of the motor tongue area. Neuroimage 20, 601–610. 10.1016/S1053-8119(03)00299-4 14527621

[B20] Finn E. Shen X. Scheinost D. Rosenberg M. Huang J. Chun M.. (2015). Functional connectome fingerprinting: identifying individuals using patterns of brain connectivity. Nat. Neurosci. 18, 1664–1671. 10.1038/nn.4135 26457551 PMC5008686

[B21] Fischl B. (2012). Freesurfer. Neuroimage 62, 774–781. 10.1016/j.neuroimage.2012.01.021 22248573 PMC3685476

[B22] Flinker A. Korzeniewska A. Shestyuk A. Franaszczuk P. Dronkers N. Knight R.. (2015). Redefining the role of Broca's area in speech. Proc. Natl. Acad. Sci. U.S.A. 112, 2871–2875. 10.1073/pnas.1414491112 25730850 PMC4352780

[B23] Friston K. J. Holmes A. P. Worsley K. J. Poline J.-P. Frith C. D. Frackowiak R. S. (1995). Statistical parametric maps in functional imaging: a general linear approach. Hum. Brain Mapp. 2, 189–210. 10.1002/hbm.460020402

[B24] Gaige T. Benner T. Wang R. Wedeen V. Gilbert R. (2007). Three dimensional myoarchitecture of the human tongue determined *in vivo* by diffusion tensor imaging with tractography. J. Magn. Reson. Imaging 26, 654–661. 10.1002/jmri.21022 17685446

[B25] Greene A. Gao S. Scheinost D. Constable R. (2018). Task-induced brain state manipulation improves prediction of individual traits. Nat. Commun. 9:2807. 10.1038/s41467-018-04920-3 30022026 PMC6052101

[B26] Greicius M. Krasnow B. Reiss A. Menon V. (2003). Functional connectivity in the resting brain: a network analysis of the default mode hypothesis. Proc. Natl. Acad. Sci. U.S.A. 100, 253–258. 10.1073/pnas.0135058100 12506194 PMC140943

[B27] Griswold M. Jakob P. Heidemann R. Nittka M. Jellus V. Wang J.. (2002). Generalized autocalibrating partially parallel acquisitions (GRAPPA). Magn. Reson. Med. 47, 1202–1210. 10.1002/mrm.10171 12111967

[B28] Haggard P. de Boer L. (2014). Oral somatosensory awareness. Neurosci. Biobehav. Rev. 47, 469–484. 10.1016/j.neubiorev.2014.09.015 25284337

[B29] Hiiemae K. Palmer J. (2003). Tongue movements in feeding and speech. Crit. Rev. Oral. Biol. Med. 14, 413–429. 10.1177/154411130301400604 14656897

[B30] Hummel T. Genow A. Landis B. (2010). Clinical assessment of human gustatory function using event related potentials. J. Neurol. Neurosurg. Psychiatry 81, 459–464. 10.1136/jnnp.2009.183699 19726416

[B31] Hyvärinen A. (1999). Fast and robust fixed-point algorithms for independent component analysis. IEEE Trans. Neural Netw. 10, 626–634. 10.1109/72.761722 18252563

[B32] Jenkinson M. Bannister P. Brady M. Smith S. (2002). Improved optimization for the robust and accurate linear registration and motion correction of brain images. Neuroimage 17, 825–841. 10.1006/nimg.2002.1132 12377157

[B33] Jenkinson M. Beckmann C. Behrens T. Woolrich M. Smith S. (2012). FSL. Neuroimage 62, 782–790. 10.1016/j.neuroimage.2011.09.015 21979382

[B34] Jiang R. Zuo N. Ford J. Qi S. Zhi D. Zhuo C.. (2020). Task-induced brain connectivity promotes the detection of individual differences in brain-behavior relationships. Neuroimage 207:116370. 10.1016/j.neuroimage.2019.116370 31751666 PMC7345498

[B35] Jungblut M. Huber W. Pustelniak M. Schnitker R. (2012). The impact of rhythm complexity on brain activation during simple singing: an event-related fMRI study. Restor. Neurol. Neurosci. 30, 39–53. 10.3233/RNN-2011-0619 22082766

[B36] Kaas J. Qi H. Iyengar S. (2006). Cortical network for representing the teeth and tongue in primates. Anat. Rec. A Discov. Mol. Cell. Evol. Biol. 288, 182–190. 10.1002/ar.a.20267 16411246

[B37] Kanai R. Rees G. (2011). The structural basis of inter-individual differences in human behaviour and cognition. Nat. Rev. Neurosci. 12, 231–242. 10.1038/nrn3000 21407245

[B38] Kennedy D. Kieser J. Bolter C. Swain M. Singh B. Waddell J. (2010). Tongue pressure patterns during water swallowing. Dysphagia 25, 11–19. 10.1007/s00455-009-9223-2 19568810

[B39] Kier W. Smith K. (1985). Tongues, tentacles and trunks: the biomechanics of movement in muscular-hydrostats. Zool. J. Linn. Soc. 83, 307–324. 10.1111/j.1096-3642.1985.tb01178.x

[B40] Kobayakawa T. Wakita M. Saito S. Gotow N. Sakai N. Ogawa H. (2005). Location of the primary gustatory area in humans and its properties, studied by magnetoencephalography. Chem. Senses 30, i226–i227. 10.1093/chemse/bjh196 15738127

[B41] Kokkonen S. Nikkinen J. Remes J. Kantola J. Starck T. Haapea M.. (2009). Preoperative localization of the sensorimotor area using independent component analysis of resting-state fMRI. Magn. Reson. Imaging 27, 733–740. 10.1016/j.mri.2008.11.002 19110394

[B42] Kollewe K. Münte T. Samii A. Dengler R. Petri S. Mohammadi B. (2011). Patterns of cortical activity differ in ALS patients with limb and/or bulbar involvement depending on motor tasks. J. Neurol. 258, 804–810. 10.1007/s00415-010-5842-7 21128080

[B43] Komoda Y. Iida T. Kothari M. Komiyama O. Baad-Hansen L. Kawara M.. (2015). Repeated tongue lift movement induces neuroplasticity in corticomotor control of tongue and jaw muscles in humans. Brain Res. 1627, 70–79. 10.1016/j.brainres.2015.09.016 26399776

[B44] Ladefoged P. Maddieson I. (1996). The Sounds of the World's Languages. Oxford: Blackwell.

[B45] Leopold N. Daniels S. (2010). Supranuclear control of swallowing. Dysphagia 25, 250–257. 10.1007/s00455-009-9249-5 19730940

[B46] Lowell S. Reynolds R. Chen G. Horwitz B. Ludlow C. (2012). Functional connectivity and laterality of the motor and sensory components in the volitional swallowing network. Exp. Brain Res. 219, 85–96. 10.1007/s00221-012-3069-9 22441258 PMC3374850

[B47] Malandraki G. Johnson S. Robbins J. (2011). Functional MRI of swallowing: from neurophysiology to neuroplasticity. Head Neck 33, S14–20. 10.1002/hed.21903 21901779 PMC3747973

[B48] Malandraki G. Sutton B. Perlman A. Karampinos D. Conway C. (2009). Neural activation of swallowing and swallowing-related tasks in healthy young adults: an attempt to separate the components of deglutition. Hum. Brain Mapp. 30, 3209–3226. 10.1002/hbm.20743 19247994 PMC6870848

[B49] Markett S. Reuter M. Montag C. Voigt G. Lachmann B. Rudorf S.. (2014). Assessing the function of the fronto-parietal attention network: insights from resting-state fMRI and the attentional network test. Hum. Brain Mapp. 35, 1700–1709. 10.1002/hbm.22285 23670989 PMC6869384

[B50] Martin R. MacIntosh B. Smith R. Barr A. Stevens T. Gati J.. (2004). Cerebral areas processing swallowing and tongue movement are overlapping but distinct: a functional magnetic resonance imaging study. J. Neurophysiol. 92, 2428–2443. 10.1152/jn.01144.2003 15163677

[B51] Martin R. Murray G. Kemppainen P. Masuda Y. Sessle B. (1997). Functional properties of neurons in the primate tongue primary motor cortex during swallowing. J. Neurophysiol. 78, 1516–1530. 10.1152/jn.1997.78.3.1516 9310440

[B52] McClung J. Goldberg S. (2002). Organization of the hypoglossal motoneurons that innervate the horizontal and oblique components of the genioglossus muscle in the rat. Brain Res. 950, 321–324. 10.1016/S0006-8993(02)03240-7 12231261

[B53] McKeown M. Jung T. Makeig S. Brown G. Kindermann S. Lee T.. (1998a). Spatially independent activity patterns in functional MRI data during the stroop color-naming task. Proc. Natl. Acad. Sci. U.S.A. 95, 803–810. 10.1073/pnas.95.3.803 9448244 PMC33801

[B54] McKeown M. Makeig S. Brown G. Jung T. Kindermann S. Bell A.. (1998b). Analysis of fMRI data by blind separation into independent spatial components. Hum. Brain Mapp. 6, 160–188. 10.1002/(SICI)1097-0193(1998)6:3<160::AID-HBM5>3.0.CO;2-1 9673671 PMC6873377

[B55] Mehl M. Vazire S. Ramírez-Esparza N. Slatcher R. Pennebaker J. (2007). Are women really more talkative than men? Science 317:82. 10.1126/science.1139940 17615349

[B56] Mohammadi B. Kollewe K. Samii A. Krampfl K. Dengler R. Münte T. (2009). Decreased brain activation to tongue movements in amyotrophic lateral sclerosis with bulbar involvement but not Kennedy syndrome. J. Neurol. 256, 1263–1269. 10.1007/s00415-009-5112-8 19353225

[B57] Monti M. (2011). Statistical analysis of fMRI time-series: a critical review of the GLM approach. Front. Hum. Neurosci. 5:28. 10.3389/fnhum.2011.00028 21442013 PMC3062970

[B58] Mu L. Sanders I. (2010). Human tongue neuroanatomy: nerve supply and motor endplates. Clin. Anat. 23, 777–791. 10.1002/ca.21011 20607833 PMC2955167

[B59] Mwangi B. Tian T. Soares J. (2014). A review of feature reduction techniques in neuroimaging. Neuroinformatics 12, 229–244. 10.1007/s12021-013-9204-3 24013948 PMC4040248

[B60] Ozdemir E. Norton A. Schlaug G. (2006). Shared and distinct neural correlates of singing and speaking. Neuroimage 33, 628–635. 10.1016/j.neuroimage.2006.07.013 16956772

[B61] Pardo J. Wood T. Costello P. Pardo P. Lee J. (1997). PET study of the localization and laterality of lingual somatosensory processing in humans. Neurosci. Lett. 234, 23–26. 10.1016/S0304-3940(97)00650-2 9347937

[B62] Pruim R. Mennes M. van Rooij D. Llera A. Buitelaar J. Beckmann C. (2015). ICA-AROMA: a robust ICA-based strategy for removing motion artifacts from fMRI data. Neuroimage 112, 267–277. 10.1016/j.neuroimage.2015.02.064 25770991

[B63] Pugnaghi M. Meletti S. Castana L. Francione S. Nobili L. Mai R.. (2011). Features of somatosensory manifestations induced by intracranial electrical stimulations of the human insula. Clin. Neurophysiol. 122, 2049–2058. 10.1016/j.clinph.2011.03.013 21493128

[B64] Reineberg A. Andrews-Hanna J. Depue B. Friedman N. Banich M. (2015). Resting-state networks predict individual differences in common and specific aspects of executive function. Neuroimage 104, 69–78. 10.1016/j.neuroimage.2014.09.045 25281800 PMC4262251

[B65] Riecker A. Ackermann H. Wildgruber D. Meyer J. Dogil G. Haider H.. (2000). Articulatory/phonetic sequencing at the level of the anterior perisylvian cortex: a functional magnetic resonance imaging (fMRI) study. Brain Lang. 75, 259–276. 10.1006/brln.2000.2356 11049668

[B66] Riecker A. Mathiak K. Wildgruber D. Erb M. Hertrich I. Grodd W.. (2005). fMRI reveals two distinct cerebral networks subserving speech motor control. Neurology 64, 700–706. 10.1212/01.WNL.0000152156.90779.89 15728295

[B67] Rudney J. Ji Z. Larson C. (1995). The prediction of saliva swallowing frequency in humans from estimates of salivary flow rate and the volume of saliva swallowed. Arch. Oral. Biol. 40, 507–512. 10.1016/0003-9969(95)00004-9 7677595

[B68] Sakamoto K. Nakata H. Inui K. Perrucci M. Del Gratta C. Kakigi R.. (2010). A difference exists in somatosensory processing between the anterior and posterior parts of the tongue. Neurosci. Res. 66, 173–179. 10.1016/j.neures.2009.10.013 19896988

[B69] Sanders I. Mu L. (2013). A three-dimensional atlas of human tongue muscles. Anat. Rec. 296, 1102–1114. 10.1002/ar.22711 23650264 PMC3687025

[B70] Sanders I. Mu L. Amirali A. Su H. Sobotka S. (2013). The human tongue slows down to speak: muscle fibers of the human tongue. Anat. Rec. 296, 1615–1627. 10.1002/ar.22755 23929762 PMC3787083

[B71] Sasaki M. Onishi K. Stefanov D. Kamata K. Nakayama A. Yoshikawa M. (2016). Tongue interface based on surface EMG signals of suprahyoid muscles. Robomech. J. 3:9 10.1186/s40648-016-0048-0

[B72] Sawczuk A. Mosier K. (2001). Neural control of tongue movement with respect to respiration and swallowing. Crit. Rev. Oral. Biol. Med. 12, 18–37. 10.1177/10454411010120010101 11349959

[B73] Schumacher S. (1927). Die Zunge, in Verdauungsapparat. Handbuch der Mikroskopischen Anatomie des Menschen, eds Hellman T. Schumacher S. Seifert E. Zimmermann K. (Berlin; Heidelberg: Springer), 35–60. 10.1007/978-3-642-51336-7_2

[B74] Shellikeri S. Green J. Kulkarni M. Rong P. Martino R. Zinman L.. (2016). Speech movement measures as markers of bulbar disease in amyotrophic lateral sclerosis. J. Speech Lang. Hear. Res. 59, 887–899. 10.1044/2016_JSLHR-S-15-0238 27679842 PMC5345561

[B75] Shen D. Cui L. Cui B. Fang J. Li D. Ma J. (2015). A systematic review and meta-analysis of the functional MRI investigation of motor neuron disease. Front. Neurol. 6:246. 10.3389/fneur.2015.00246 26635722 PMC4656846

[B76] Shinagawa H. Ono T. Ishiwata Y. Honda E. Sasaki T. Taira M.. (2003). Hemispheric dominance of tongue control depends on the chewing-side preference. J. Dent. Res. 82, 278–283. 10.1177/154405910308200407 12651931

[B77] Simonyan K. Fuertinger S. (2015). Speech networks at rest and in action: interactions between functional brain networks controlling speech production. J. Neurophysiol. 113, 2967–2978. 10.1152/jn.00964.2014 25673742 PMC4416556

[B78] Small D. (2010). Taste representation in the human insula. Brain Struct. Funct. 214, 551–561. 10.1007/s00429-010-0266-9 20512366

[B79] Smith S. (2002). Fast robust automated brain extraction. Hum. Brain Mapp. 17, 143–155. 10.1002/hbm.10062 12391568 PMC6871816

[B80] Smith S. Fox P. Miller K. Glahn D. Fox P. Mackay C.. (2009). Correspondence of the brain's functional architecture during activation and rest. Proc. Natl. Acad. Sci. U.S.A. 106, 13040–13045. 10.1073/pnas.0905267106 19620724 PMC2722273

[B81] Smith S. Jenkinson M. Woolrich M. Beckmann C. Behrens T. Johansen-Berg H.. (2004). Advances in functional and structural MR image analysis and implementation as FSL. Neuroimage 23, S208–S219. 10.1016/j.neuroimage.2004.07.051 15501092

[B82] Sörös P. Bose A. Sokoloff L. Graham S. Stuss D. (2011). Age-related changes in the functional neuroanatomy of overt speech production. Neurobiol. Aging 32, 1505–1513. 10.1016/j.neurobiolaging.2009.08.015 19782435 PMC4896807

[B83] Sörös P. Hoxhaj E. Borel P. Sadohara C. Feige B. Matthies S.. (2019). Hyperactivity/restlessness is associated with increased functional connectivity in adults with ADHD: a dimensional analysis of resting state fMRI. BMC Psychiatry 19:43. 10.1186/s12888-019-2031-9 30683074 PMC6347794

[B84] Sörös P. Inamoto Y. Martin R. (2009). Functional brain imaging of swallowing: an activation likelihood estimation meta-analysis. Hum. Brain Mapp. 30, 2426–2439. 10.1002/hbm.20680 19107749 PMC6871071

[B85] Sörös P. Lalone E. Smith R. Stevens T. Theurer J. Menon R.. (2008). Functional MRI of oropharyngeal air-pulse stimulation. Neuroscience 153, 1300–1308. 10.1016/j.neuroscience.2008.02.079 18455883

[B86] Sörös P. Macintosh B. Tam F. Graham S. (2010). fMRI-compatible registration of jaw movements using a fiber-optic bend sensor. Front. Hum. Neurosci. 4:24. 10.3389/fnhum.2010.00024 20463865 PMC2868298

[B87] Sörös P. Sokoloff L. Bose A. McIntosh A. Graham S. Stuss D. (2006). Clustered functional MRI of overt speech production. Neuroimage 32, 376–387. 10.1016/j.neuroimage.2006.02.046 16631384

[B88] Svoboda K. Li N. (2018). Neural mechanisms of movement planning: motor cortex and beyond. Curr. Opin. Neurobiol. 49, 33–41. 10.1016/j.conb.2017.10.023 29172091

[B89] Takai O. Brown S. Liotti M. (2010). Representation of the speech effectors in the human motor cortex: somatotopy or overlap. Brain Lang. 113, 39–44. 10.1016/j.bandl.2010.01.008 20171727

[B90] Tourville J. Nieto-Castañón A. Heyne M. Guenther F. (2019). Functional parcellation of the speech production cortex. J. Speech Lang. Hear. Res. 62, 3055–3070. 10.1044/2019_JSLHR-S-CSMC7-18-0442 31465713 PMC6813033

[B91] Tremblay P. Sato M. Deschamps I. (2017). Age differences in the motor control of speech: an fMRI study of healthy aging. Hum. Brain Mapp. 38, 2751–2771. 10.1002/hbm.23558 28263012 PMC6866863

[B92] Turesky T. Turkeltaub P. Eden G. (2016). An activation likelihood estimation meta-analysis study of simple motor movements in older and young adults. Front. Aging Neurosci. 8:238. 10.3389/fnagi.2016.00238 27799910 PMC5065996

[B93] Turner B. Paul E. Miller M. Barbey A. (2018). Small sample sizes reduce the replicability of task-based fMRI studies. Commun. Biol. 1:62. 10.1038/s42003-018-0073-z 30271944 PMC6123695

[B94] Tustison N. Cook P. Klein A. Song G. Das S. Duda J.. (2014). Large-scale evaluation of ANTs and FreeSurfer cortical thickness measurements. Neuroimage 99, 166–179. 10.1016/j.neuroimage.2014.05.044 24879923

[B95] Tustison N. J. Avants B. B. Cook P. A. Zheng Y. Egan A. Yushkevich P. A.. (2010). N4ITK: improved N3 bias correction. IEEE Trans. Med. Imaging 29:1310. 10.1109/TMI.2010.2046908 20378467 PMC3071855

[B96] Van Lieshout P. Steele C. Lang A. (2011). Tongue control for swallowing in Parkinson's disease: effects of age, rate, and stimulus consistency. Mov. Disord. 26, 1725–1729. 10.1002/mds.23690 21542018

[B97] Veale J. (2014). Edinburgh Handedness Inventory–Short Form: a revised version based on confirmatory factor analysis. Laterality 19, 164–177. 10.1080/1357650X.2013.783045 23659650

[B98] Watanabe J. Sugiura M. Miura N. Watanabe Y. Maeda Y. Matsue Y.. (2004). The human parietal cortex is involved in spatial processing of tongue movement–an fMRI study. Neuroimage 21, 1289–1299. 10.1016/j.neuroimage.2003.10.024 15050556

[B99] Weirich M. Lancia L. Brunner J. (2013). Inter-speaker articulatory variability during vowel-consonant-vowel sequences in twins and unrelated speakers. J. Acoust. Soc. Am. 134, 3766–3780. 10.1121/1.4822480 24180787

[B100] Wexler B. Fulbright R. Lacadie C. Skudlarski P. Kelz M. Constable R.. (1997). An fMRI study of the human cortical motor system response to increasing functional demands. Magn. Reson. Imaging 15, 385–396. 10.1016/S0730-725X(96)00232-9 9223039

[B101] Wong D. Dzemidzic M. Talavage T. Romito L. Byrd K. (2011). Motor control of jaw movements: an fMRI study of parafunctional clench and grind behavior. Brain Res. 1383, 206–217. 10.1016/j.brainres.2011.01.096 21295015

[B102] Woolrich M. Behrens T. Beckmann C. Jenkinson M. Smith S. (2004). Multilevel linear modelling for FMRI group analysis using Bayesian inference. Neuroimage 21, 1732–1747. 10.1016/j.neuroimage.2003.12.023 15050594

[B103] Woolrich M. Jbabdi S. Patenaude B. Chappell M. Makni S. Behrens T.. (2009). Bayesian analysis of neuroimaging data in FSL. Neuroimage 45, S173–S186. 10.1016/j.neuroimage.2008.10.055 19059349

[B104] Woolrich M. Ripley B. Brady M. Smith S. (2001). Temporal autocorrelation in univariate linear modeling of FMRI data. Neuroimage 14, 1370–1386. 10.1006/nimg.2001.0931 11707093

[B105] Xu A. Larsen B. Baller E. B. Scott J. C. Sharma V. Adebimpe A. (2019). Convergent neural representations of acute nociceptive pain in healthy volunteers: a large-scale fMRI meta-analysis. bioRxiv 779280 10.1101/779280 PMC7755074 31954149

[B106] Yeo B. Krienen F. Sepulcre J. Sabuncu M. Lashkari D. Hollinshead M.. (2011). The organization of the human cerebral cortex estimated by intrinsic functional connectivity. J. Neurophysiol. 106, 1125–1165. 10.1152/jn.00338.2011 21653723 PMC3174820

[B107] Ziegler W. (2003). Speech motor control is task-specific: evidence from dysarthria and apraxia of speech. Aphasiology 17, 3–36. 10.1080/729254892

[B108] Zuo X. Xing X. (2014). Test-retest reliabilities of resting-state FMRI measurements in human brain functional connectomics: a systems neuroscience perspective. Neurosci. Biobehav. Rev. 45, 100–118. 10.1016/j.neubiorev.2014.05.009 24875392

